# A SYSTEMATIC REVIEW OF RESILIENCE IN FAMILY CAREGIVERS OF AUTISTIC ADULTS: IMPLICATIONS FOR RESEARCH AND PRACTICE

**DOI:** 10.1093/geroni/igae098.3779

**Published:** 2024-12-31

**Authors:** Daphne Chakurian, Lori Popejoy

**Affiliations:** University of Missouri - Columbia, Columbia, Missouri, United States; University of Missouri - Columbia, Columbia, Missouri, United States

## Abstract

Family caregivers (FCGs) of autistic adults provide lifelong care and experience chronic stress, stigma, and social isolation, placing them at risk of poor health, quality of life, and well-being. Resilience, stress, and coping research have primarily been limited to FCGs of autistic children with inconsistent use of theory, definition, and measurement. This systematic review aimed to examine resilience in FCGs of autistic adults. Guided by the Transactional Model of Stress and Coping, this review followed PRISMA guidelines, and the protocol was registered with PROSPERO. Comprehensive searches of 10 databases identified 1,935 records. Risk of bias was appraised using the Johns Hopkins Evidence-Based Practice Model and Guide. Nineteen studies meeting inclusion criteria were included in the narrative analysis and were mapped to the conceptual model. Designs were cross-sectional and phenomenological. We found that higher FCG quality of life and well-being was associated with increased problem and meaning-focused coping, social support, future planning, and less perceived burden and stigma. A variety of FCG and autistic adult person and environment factors provided context for the stress and coping process. Social support and coping strategies were key to FCG resilience outcomes and may inform clinical guidelines and policy development. Research is warranted to examine social support, coping strategy, future planning, and perceived burden and stigma as modifiable factors to enhance FCG resilience. The use of well-defined theoretical constructs and measures could facilitate future systematic reviews, as well as contribute to clinical guidelines and policy development.

